# The Histone Deacetylase HDAC4 Regulates Long-Term Memory in *Drosophila*

**DOI:** 10.1371/journal.pone.0083903

**Published:** 2013-12-09

**Authors:** Helen L. Fitzsimons, Silvia Schwartz, Fiona M. Given, Maxwell J. Scott

**Affiliations:** 1 Institute of Fundamental Sciences, Massey University, Palmerston North, New Zealand; 2 Department of Entomology, North Carolina State University, Raleigh, North Carolina, United States of America; Alexander Fleming Biomedical Sciences Research Center, Greece

## Abstract

A growing body of research indicates that pharmacological inhibition of histone deacetylases (HDACs) correlates with enhancement of long-term memory and current research is concentrated on determining the roles that individual HDACs play in cognitive function. Here, we investigate the role of HDAC4 in long-term memory formation in *Drosophila*. We show that overexpression of HDAC4 in the adult mushroom body, an important structure for memory formation, resulted in a specific impairment in long-term courtship memory, but had no affect on short-term memory. Overexpression of an HDAC4 catalytic mutant also abolished LTM, suggesting a mode of action independent of catalytic activity. We found that overexpression of HDAC4 resulted in a redistribution of the transcription factor MEF2 from a relatively uniform distribution through the nucleus into punctate nuclear bodies, where it colocalized with HDAC4. As MEF2 has also been implicated in regulation of long-term memory, these data suggest that the repressive effects of HDAC4 on long-term memory may be through interaction with MEF2. In the same genetic background, we also found that RNAi-mediated knockdown of HDAC4 impairs long-term memory, therefore we demonstrate that HDAC4 is not only a repressor of long-term memory, but also modulates normal memory formation.

## Introduction

Formation of long-term memory (LTM) requires acetylation of specific histone residues at the promoters of plasticity-associated genes, the catalysis of which is governed by the opposing activities of histone acetyltransferases (HATs) and histone deacetylases (HDACs). Disruption of this balance can result in impairment of LTM, for instance, mice heterozygous for a knockout of the CREB binding protein (CBP), a HAT, display deficits in associative memory [1], as do mice with focal knockouts of CBP in the hippocampus [2]. Similarly, mice with disrupted HAT activity via a dominant negative form of CBP also have impaired memory [3]. A reciprocal effect on histone acetylation has also been demonstrated by treatment with HDAC inhibitors during training. Systemic administration of the HDAC inhibitors sodium butyrate (NaBu), trichostatin A or suberoylanilide hydroxamic acid (SAHA) enhances formation of LTM in rodent memory tasks [1,4–7], and mice trained to a sub-threshold level form a long-lasting stable memory when NaBu is administered immediately after training [8]. Inhibition of HDAC activity also rescues memory impairments in rodent models of neurodegenerative disease [1,6,9,10].

The above-described HDAC inhibitors are relatively non-specific, and recent efforts have concentrated on identifying the specific HDACs that are responsible for repression of memory, which will improve understanding of the molecular processes by which memory formation is regulated, as well as identifying specific HDACs to target for drug development. There are eleven zinc-dependent HDACs in vertebrates, which can be separated into three classes based on their sequence similarity to yeast Hda1. Class I HDACs (HDAC1, 2, 3 and 8) are generally localized to the nucleus and data from several studies suggest that the enhancement of LTM by HDAC inhibitors is mediated through inhibition of HDAC2 and 3 [11–13]. Overexpression of HDAC2 in the mouse brain decreases dendritic spine formation and impairs memory formation, both of which are rescued by chronic administration of SAHA. Conversely, knockout of HDAC2 enhances both STM and LTM, whereas overexpression of HDAC1 has no effect on memory, indicating that the SAHA-induced memory enhancement is likely mediated through inhibition of HDAC2 [12]. Short-hairpin-RNA mediated knockdown of HDAC2 expression also rescues neurodegeneration-induced memory impairments in a mouse model of Alzheimer’s disease-related pathologies [11]. Negative regulation of LTM is not only mediated through the activity of HDAC2, however, as focal knockout of HDAC3 in the CA1 region of the adult mouse hippocampus also enhances LTM, as does treatment with a selective HDAC3 inhibitor [13]. Moreover, regulation of LTM by Class I HDACs is conserved across animal kingdom, as overexpression of Rpd3 (which has equal homology to human HDAC1 and 2) in the adult *Drosophila melanogaster* brain also impairs LTM, without having any impact on STM [14]. The six Class II HDACs can be separated into two groups: Class IIa HDACs (HDAC4, 5, 7 and 9) exhibit nucleocytoplasmic shuttling in response to physiological stimuli [15,16], whereas the Class IIb HDACs (HDAC6 and 10), are exclusively cytoplasmic [17,18]. There is a single Class III HDAC, HDAC11, which is expressed in the brain [19] and little is known about its function.

Perhaps because current HDAC inhibitors almost exclusively target Class I HDACs [20], there has been relatively little research on the role of Class IIa HDACs in memory. However a body of evidence is accumulating to suggest that Class IIa HDACs also play a role in regulation of memory formation. Class IIa HDAC4 are expressed in the mammalian brain [21], and a comprehensive analysis of HDAC4 expression in the mouse brain has been performed, which revealed widespread expression throughout the brain including the hippocampus and cortex [22]. Within the hippocampus, a brain region that plays a key role in learning and memory in mammals, HDAC4 immunoreactivity is present in the cytoplasm of most neurons but only in a subset of nuclei, reflecting differential regulation of subcellular localization. Of particular interest, a high number of immunoreactive puncta are present along dendritic shafts, with the strongest accumulation at the post-synaptic density [22], a structure critical for memory formation. Nucleocytoplasmic shuttling of HDAC4 occurs in response to Ca^2+^ influx [16] and is regulated by phosphorylation. Kinases responsible include CaMKII [15,23], a putative synaptic tag, which is a molecule that labels synapses for capturing of plasticity-related proteins [24]. Phosphorylation of HDAC4 creates docking sites for the chaperone 14-3-3, which then escorts it from nucleus [25,26].

The most well characterised function of Class IIa HDACs is repression of transcription, which occurs via binding to transcriptional activators rather than through their deacetylase activity, which is very weak in comparison to Class I HDACs [27]. One such target is the MEF2 family of transcription factors, which influence a myriad of cellular functions by controlling transcriptional programs. HDAC4 binds MEF2, which leads to repression of MEF2 activity and negative regulation of developmental programs such as myoblast differentiation [28] and chondrocyte hypertrophy [29,30]. Interestingly, recent studies have demonstrated a critical role for MEF2 family members in learning and memory. Brain-specific knockout of MEF2C in the mouse hippocampus impairs contextual fear conditioning [31], and expression of a constitutively active form of MEF2 in the mouse hippocampal and amygdala also blocks both spatial and fear memory, respectively [32]. Taken together, these studies paint a compelling picture that Class IIa HDACs play an integral role in brain function, and furthermore, in considering the association of Class II HDACs with other memory proteins, we hypothesised that Class IIa HDACs activity would regulate LTM formation. Indeed during the course of this work, two studies demonstrating a critical role of HDAC4 in rodent memory formation were described. Expression of a truncated, nuclear-restricted HDAC4 mutant impairs spatial memory in mice and represses a set of genes involved in synaptic plasticity [33]. A brain-specific knockout of HDAC4 also impairs LTP and hippocampal-dependent memory also in mice [34]. Loss of HDAC4 also represses thermosensation memory in *C. elegans* [35]. The observations that nuclear-restricted HDAC4 can inhibit LTM, yet a depletion of HDAC4 also impairs LTM, suggest the role of HDAC4 in regulation of memory is complex, and warrants further investigation.

We sought to further examine role of HDAC4 in memory in the model genetic organism *D. melanogaster*. *Drosophila* is an excellent model for molecular dissection of memory processes, foremost for its tractability to genetic manipulation [36,37] as well as the reproducible assays that have been developed for evaluation of memory (for review see 38). HDACs are highly conserved across species and the *Drosophila* genome contains five zinc-dependent HDACs: Rpd3, HDAC3, HDAC4, HDAC6 and HDAC11 [39]. We previously reported that the Class I HDAC Rpd3 plays a critical role in LTM [14]. *Drosophila* contains a single Class IIa HDAC, HDAC4, the roles of which in brain function or memory have not been characterised. We report here that HDAC4 plays an important role in modulating long-term courtship memory. Overexpression of HDAC4 in the mushroom body impairs LTM, but not STM, and this effect is independent of its deacetylase activity. Moreover, we demonstrate that wild-type HDAC4 is required for long-term conditioning of male courtship behavior, as RNAi-mediated knockdown of HDAC4 also results in impairment of LTM. 

## Results

Sequence elements essential for vertebrate HDAC4 function are conserved in *Drosophila.*

**Figure 1:**
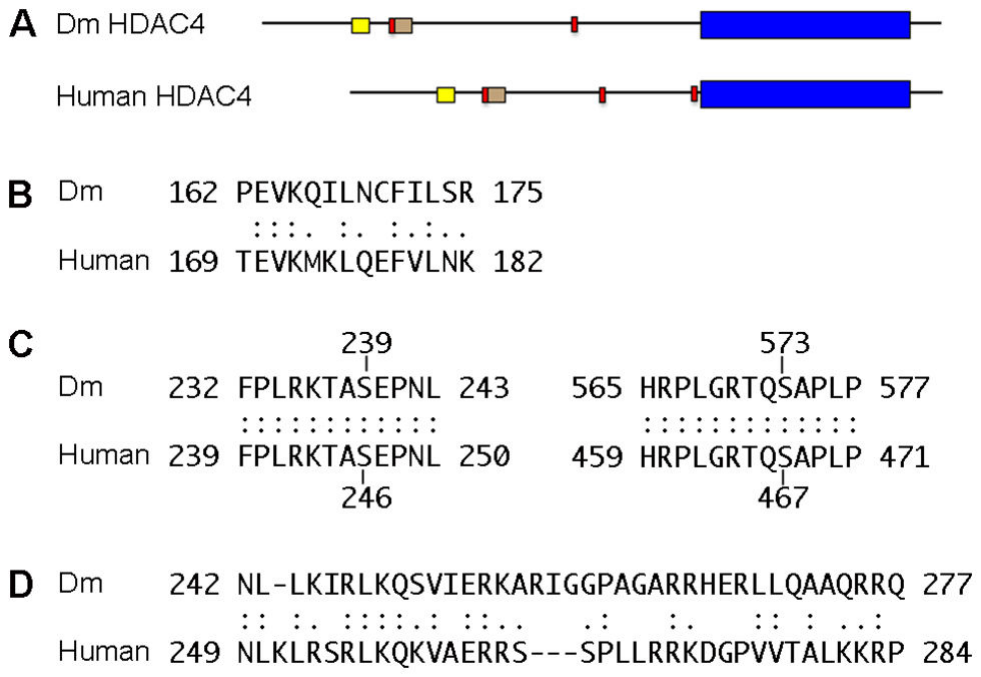
Domain organisation of human and Drosophila HDAC4 proteins. A. One isoform of each protein is shown (Human HDAC4 GenBank accession NP_006028, 1084 amino acids; Drosophila HDAC4, isoform D, GenBank accession NP_572868, 1252 amino acids). Regions of human HDAC4 essential for function and corresponding regions of Drosophila HDAC4 are depicted. The deacetylase domain is shown in blue, MEF2 binding motif in yellow, 14-3-3 association sites in red, and the nuclear import signal in brown. B-C. Sequence alignment of Drosophila HDAC4 with regions of human HDAC4 essential for function. B. MEF2 binding motif. C. Conserved serine residues required for 14-3-3ζ binding are shown. D. The residues identified to mediate nuclear localization of vertebrate HDAC4 are also highly conserved.

*Drosophila* HDAC4 is relatively highly conserved, with 57% amino acid identity and 84% similarity to human HDAC4 across the deacetylase domain-containing C terminus, and 35% identity and 59% similarity across the whole protein (Figure 1A). The N terminus of Class II HDACs contains elements critical for function and regulation of subcellular localization, which are conserved in Drosophila HDAC4 and include a MEF2 binding motif (Figure 1B) serine residues for 14-3-3 mediated nuclear export (Figure 1C) and a putative nuclear import signal (Figure 1D).

### HDAC4-trapped p[GAL4] drives expression in the mushroom body (MB)

**Figure 2:**
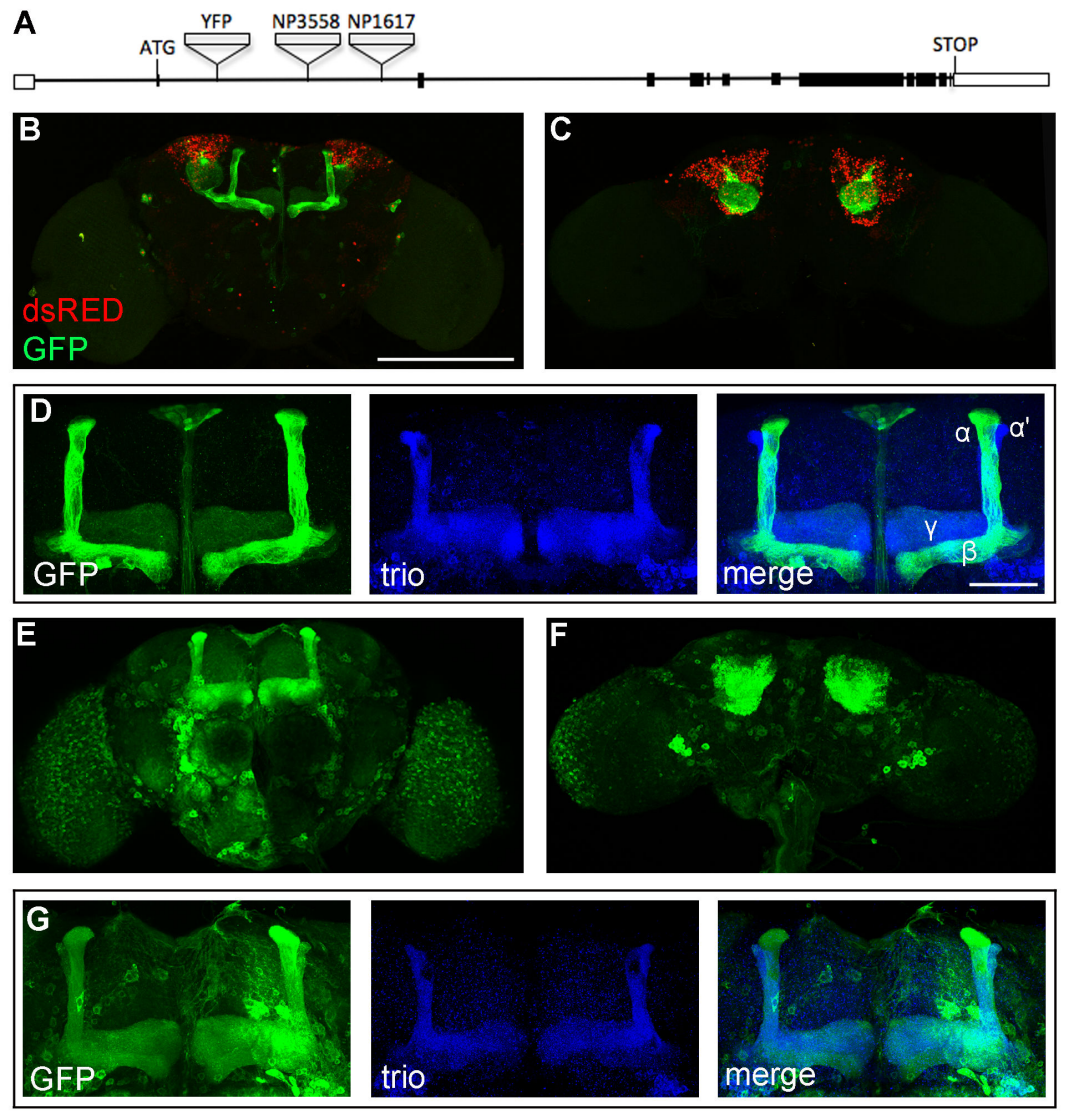
Characterisation of HDAC4-trapped p{GAL4}-driven expression. A. Molecular organisation of the HDAC4 gene locus. The 22.6 kb locus is shown, with exons depicted as boxes. Translated regions are black boxes. The P element transposons NP3558 and NP1617 are inserted 3144 and 4838 bp downstream from the 3' end of the 2^nd^ exon, respectively. The site of YFP insertion into HDAC4::YFP at 1310 bp downstream from the 3' end of the 2^nd^ exon is also shown. YFP is flanked by splice sites, which facilitate internal incorporation of YFP into the HDAC4 protein. B-D. Confocal microscopy images of whole mount brains with NP1617-driven expression of nls.dsRED and CD8::GFP. B. Frontal confocal projection through the brain showing CD8::GFP in the MB and nls.dsRED in the Kenyon cell nuclei. Some expression can be seen at lower levels in other regions of the brain. Scale bar = 200 μm for B,C,E,F. C. Posterior view showing CD8::GFP localized to the calyx and nls.dsRED in the Kenyon cell nuclei. D. CD8::GFP expression is observed in α/β and γ lobes of the MB. Immunohistochemistry against Trio (blue) which is expressed in the α'/β' and γ lobes of the mushroom body, confirms co-localization of GFP with Trio in the γ but not the α'/β' lobes. Scale bar = 50 μm for D and G. E-G. Confocal microscopy images of whole mount brains with NP3558-driven expression of CD8::GFP. E. Frontal confocal projection showing CD8::GFP in the mushroom body and widespread through the rest of the brain. F. Posterior view showing high expression of GFP in the Kenyon cells. G. Anti-Trio immunohistochemistry reveals colocalization of GFP and Trio in the α'/β' and γ lobes, and GFP is also observed in the Trio-negative α/β lobes.

We first sought to characterize the endogenous expression pattern of HDAC4 in the adult Drosophila brain, which has not yet been reported. As no Drosophila HDAC4 antibody was available, and nor were we successful in generating one, we took advantage of two p{GawB} enhancer trap lines that are inserted into the HDAC4 locus and therefore in the vicinity of enhancers that drive expression of HDAC4. The NP1617 and NP3558 P-element transposons are inserted in the 2^nd^ intron of the HDAC4 gene, 4838 and 3144 bp downstream from the 3' end of the 2^nd^ exon, respectively (isoform HDAC4-RD, Figure 2A). The next closest gene is >7 kb from the P-element insertions. To examine the expression pattern driven by the two p[GAL4] lines, we crossed each of them to a line harbouring UAS-CD8::GFP and UAS-Redstinger. Redstinger is a nuclear localized dsRED (nls.dsRED) and CD8::GFP is a plasma membrane-targeted GFP, which together allow for visualisation of GFP in neuronal processes that surround the dsRED-filled nucleus. NP1617 drove expression primarily in the MB, with minimal expression elsewhere in the brain (Figure 2B,C). The MB is a structure critical for memory formation in Drosophila [40–42]. The intrinsic neurons of the MB are the Kenyon cells, of which there are three specific classes (α/β, α'/β' and γ). The axons of the three neuronal subtypes are bundled to together to form a ventrally projecting peduncle, which then splits to form lobes. The α/β and α'/β' axons both bifurcate to form the vertical α and α' lobes and the medial β and β' lobes, while the γ neuron axons form a single medial lobe. NP1617 drove expression in the α/β and γ lobes (Figure 2D), but not the α'/β' lobes, as shown by a lack of co-expression in these lobes with Trio, which is expressed in α'/β' and γ neurons. NP3558-driven expression of nls.dsRED caused lethality at the pupal stage, therefore the expression pattern driven by NP3558 was characterised using CD8::GFP only (Figure 2E, F). The expression level achieved with this driver was much higher than NP1617, with CD8::GFP protein present in all lobes of the MB (Figure 2G) and widespread throughout the brain. Notably, the expression NP1617 and NP3558 pattern overlapped in the MB. The lack of NP1617-driven expression in the α'/β' neurons suggests that the enhancer elements for the α/β and γ neurons may be distinct from those that drive expression in the α'/β' neurons. 

In a separate approach to examine endogenous HDAC4 expression, a fly strain harbouring an HDAC4-trapped EYFP generated by the Cambridge Protein Trap Project was obtained [43]. This consists of an artificial exon containing the EYFP gene flanked by splice acceptor and donor sequences, which is inserted into the 2^nd^ intron of the HDAC4 gene, 1310 bp downstream from the 3' end of the 2^nd^ exon (Figure 2A). This results in an internal incorporation of EYFP into the HDAC4 protein. The normal viability of the flies suggests that insertion of EYFP does not appear to inactivate or severely reduce endogenous HDAC4, in contrast to some hypomorphs of HDAC4 that are hemizygous lethal due to a role in embryo development [46]. EYFP expression was extremely low and we could not detect it by confocal microscopy on whole mount brains without amplifying the signal via immunohistochemistry with an anti-GFP antibody (which equally detects EYFP as the two proteins share the same antigen). Although the level of expression in whole-mount brains was low and difficult to distinguish from background, we did observe a weak signal in the MB that was never observed in control brains (Figure S1), which is in agreement with the MB expression driven by the two enhancer trap lines.

### Characterisation of HDAC4 overexpression in the mushroom body

**Figure 3:**
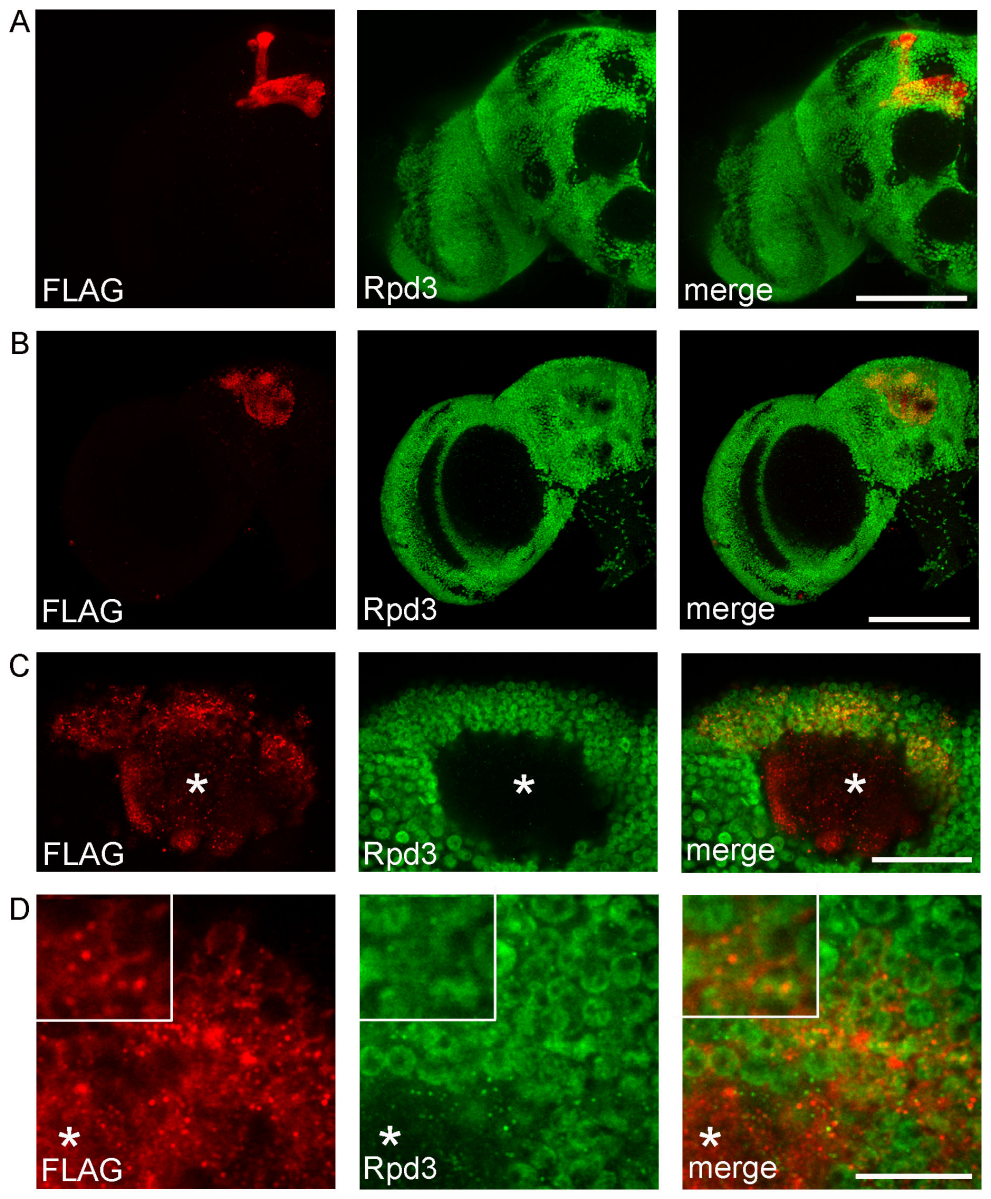
Overexpression of FLAG-tagged HDAC4 in the fly brain. OK107-GAL4 mediated expression FLAG-HDAC4 expression was induced in adult brains with the TARGET system at 30°C for three days. Whole-mount brains were processed for immunohistochemistry with anti-FLAG (red) and anti-Rpd3 (green). Rpd3 is expressed in the majority of neuronal nuclei and assists in visualization of general anatomy of the brain. A. Frontal confocal projection through one hemisphere of the brain showing localization of HDAC4 to the lobes of the MB. Scale bar = 100 μm. B. Posterior confocal projection through one hemisphere of the brain showing HDAC4 expression in the calyx and Kenyon cell cytoplasm and nuclei. Scale bar = 100 μm. C. A single confocal plane (1 μm) through the MB at the level of the calyx shows Rpd3 is localized to Kenyon cell nuclei whereas HDAC4 is predominantly localized to the calyx (asterisk) and cytoplasm. Scale bar = 25 μm. D. Magnification of same region showing higher definition of Kenyon cell expression. Inset - a cytoplasmic halo around the nucleus and punctate nuclear expression can be seen in a subset of nuclei. Scale bar = 10 μm. Inset in D is at 2x magnification.

As long-term courtship memory is dependent on an intact MB [41], we chose to examine importance of HDAC4 in LTM by modulating HDAC4 levels in the MB and assessing the effect on LTM using the courtship suppression assay. We generated transgenic flies containing an N-terminus FLAG-tagged HDAC4 gene driven by a UAS_G_-hsp70_min_ enhancer-promoter. Expression was induced in the adult fly brain using the GAL4 driver OK107. We selected this driver for its ability to facilitate high expression in Kenyon cells, the intrinsic neurons of the MB [14,44,45]. Drosophila HDAC4 plays a role in embryo segmentation [46], therefore in order to obviate potential effects of HDAC4 overexpression on development, we restricted HDAC4 expression to adult brains with the temporal and regional gene expression targeting (TARGET) system [37]. In this system, flies are raised at 19°C, at which GAL4-mediated gene expression is inhibited by a temperature sensitive mutant of GAL80 (GAL80ts). When the temperature is raised to 30°C, GAL80ts is inactivated and transgene expression ensues. To aid visualisation of the general anatomy of the brain, whole-mount brains were subjected to immunohistochemistry with an antibody to the Class I HDAC Rpd3, which is expressed in all neuronal nuclei [14], and co-labelled with anti-FLAG to detect HDAC4. In contrast to the nuclear Rpd3, the majority of HDAC4 was localized to the MB lobes (Figure 3A and Figure S2). HDAC4 was not detected in the brains of flies maintained at 19°C (Figure S3). HDAC4 was also localized to the calyx, the dendritic field of the Kenyon cells (Figure 3B, C), and in a subset of Kenyon cell nuclei. Within the nuclei, HDAC4 was localized to discrete regions, appearing as punctate nuclear bodies (Figure 3D), which have been previously observed when HDAC4 is expressed in cultured cells [47,48].

### Overexpression of HDAC4 impairs LTM

The repeat training courtship assay was used to assess 24 hour LTM. This assay measures the ability of a male fly to remember that his advances were previously rejected by a female. Wild-type flies subjected to a seven-hour training session form a robust LTM that is stable for at least 24 hours [14,49]. Memory is compared between groups by calculation of a memory index (MI), which is calculated by dividing the courtship index (CI) of each test fly by the mean CI of the sham control flies (CI_trained_/mCI_sham_), allowing comparison of memory between genotypes [50–52]. A score of 0 indicates the highest memory performance possible, and a score of ≥1.0 indicates no memory

**Figure 4:**
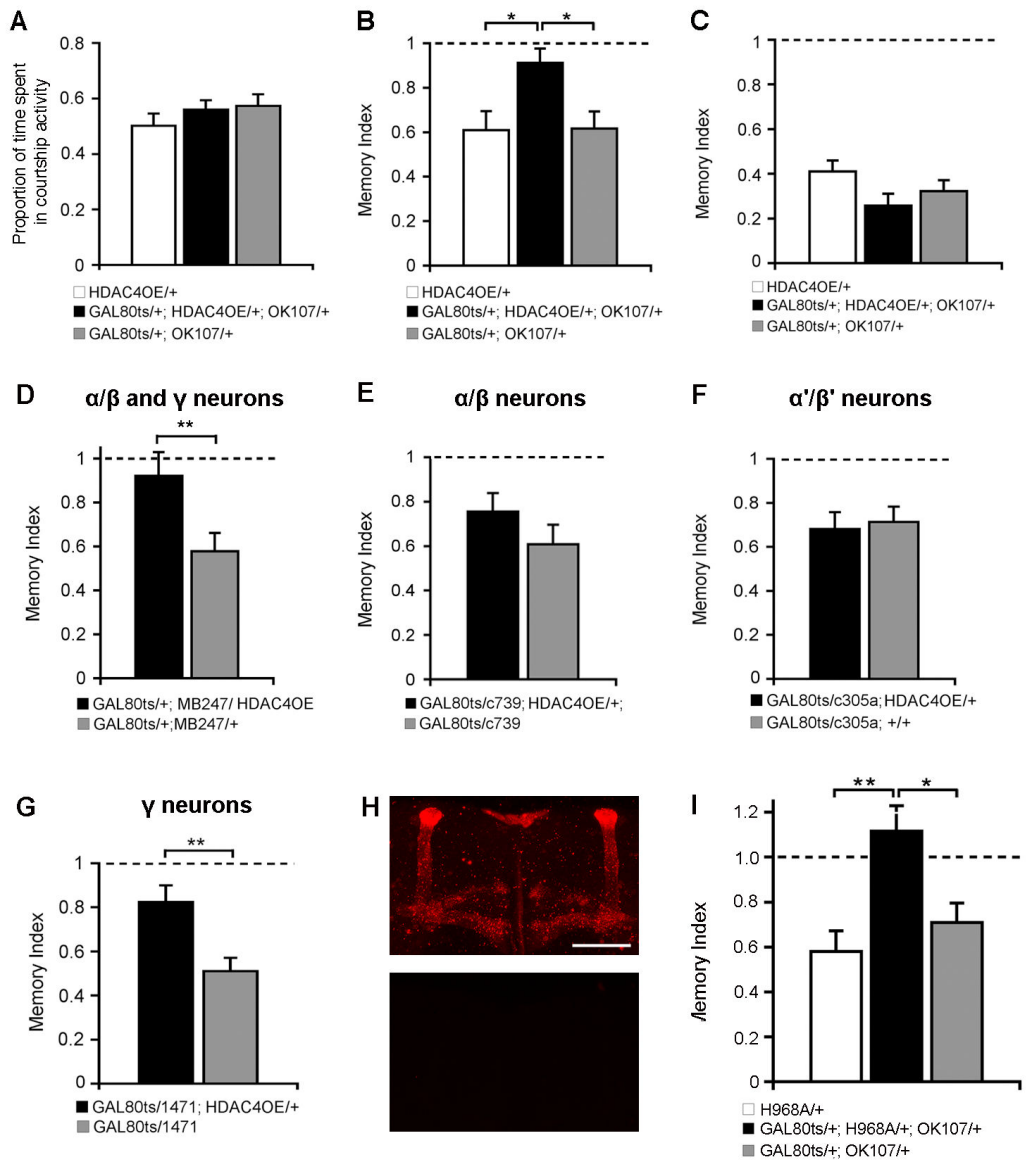
Overexpression of HDAC4 impairs LTM. In all cases, HDAC4 expression was restricted to the adult brain with the TARGET system. A, B. Courtship activity of naïve males was not altered by OK107-GAL4 mediated overexpression of HDAC4 (ANOVA, p=0.377) (A), but LTM was significantly impaired (ANOVA, post-hoc Tukey’s HSD, *p<0.05) (B). C. STM tested one hour following a one-hour training session was not significantly different between control and HDAC4-overexpressing males (ANOVA, p=0.12). D. Overexpression with α/β and γ lobe driver MB247 significantly impaired LTM (Student’s t-test, two-tailed, unpaired, **p<0.01). E. No significant impairment in LTM occurred when MB expression of HDAC4 was restricted to the α/β lobes with the c739 driver (Student’s t-test, two-tailed, unpaired, p=0.156). F. LTM was also not impaired when FLAG-HDAC4 was expressed in the α'/β' lobes with the c305a driver (Student’s t-test, two-tailed, unpaired, p=0.630), G. 1471-GAL4 driven expression of FLAG-HDAC4 in the γ lobe resulted in a significant LTM deficit (Student’s t-test, two-tailed, unpaired, **p<0.01). H. OK107-GAL4 mediated expression of FLAG-HDAC4 H968A was induced in adult brains with the TARGET system. Whole-mount brains were processed for immunohistochemistry against FLAG, which detects HDAC4 H968A in the mushroom body (upper image). No expression was observed when flies were raised and maintained at 19°C (lower image). Scale bar = 50 μm. I. Expression of HDAC4 H986A in the MB impaired LTM (ANOVA, post-hoc Tukey’s HSD *p<0.5, **p<0.05).

To restrict expression of HDAC4 to adulthood, flies were raised at 19°C and then after eclosion, males were collected and transferred to 30°C for three days prior to training to allow induction of HDAC4 expression. Males of all genotypes displayed the repertoire of normal courtship behaviours and there was no difference between groups in naïve courtship activity (Figure 4A). Control males also retained normal LTM, whereas those in which HDAC4 was overexpressed displayed in a significant impairment (Figure 4B). To examine whether HDAC4 regulates an earlier phase of memory, short-term memory (STM) was assessed by subjecting males to a one-hour training session and then testing one hour later. Males of all genotypes displayed normal STM (Figure 4C), thus increased HDAC4 had no impact on one-hour memory.

Although the highest expression driven by OK107-GAL4 is in the MB, it does drive lower expression in other regions of the brain including the pars intercerebralis, suboesophageal ganglion and optic lobes [14,44].. To further examine whether the observed negative effect of HDAC4 on memory is mediated by the MB, we repeated the memory assay using the MB247 driver, which drives robust expression in the α/β and γ lobes of the MB, but little else in the brain except for very low levels in the ellipsoid body, lobula plate of the optic lobe and glia, [44,53]. MB247-driven expression of HDAC4 also impaired LTM (Figure 4D), therefore MB-specific overexpression of HDAC4 is the likely root of the memory impairment.

We examined the contribution of α/β, α'/β' and γ neurons to HDAC4-mediated impairment of LTM by restricting expression of HDAC4 to each of these Kenyon cell subtypes with specific GAL4 drivers. In all experiments, expression was induced in adult males with GAL80ts. The GAL4 drivers c739 and c305a facilitate expression in the α/β and α'/β' Kenyon cell subtypes, respectively [44,54,55]. Overexpression of HDAC4 with either of these drivers did not have a significant effect on LTM (Figure 4E,F), however, expression of HDAC4 in γ neurons with the 1471 driver [56] recapitulated the impairment on LTM that was observed with OK107 and MB247 (Figure 4G). c739, c305a and 1471 all drive expression in the brain outside of the MB, albeit at lower levels. The non-MB expression driven by 1471 overlaps largely with that of c305a and c739, suggesting that expression of HDAC4 in these brain areas does not contribute to the memory deficit and the only brain region that always correlates with impairment of LTM is the γ lobe. Taken together, these data suggesting that the negative effect of HDAC4 on courtship suppression is mediated through the γ neurons. 

The deacetylase activity of HDAC4 is dispensable for some physiological functions and vertebrate HDAC4 possesses little activity [27,57,58]. We generated transgenic flies harbouring a catalytically impaired HDAC4 cDNA by replacing the histidine at position 968 with an alanine. This histidine residue (corresponding to human H803) is conserved across vertebrates and invertebrates and is critical for function at the active site and mutation of this residue severely attenuates catalytic activity [59–61]. This construct was otherwise identical to FLAG-HDAC4 and its expression pattern in the brain was also indistinguishable from that of HDAC4 (Figure 4H). Overexpression of HDAC4 H968A in the adult fly brain abolished LTM (Figure 4I), thus the capacity of HDAC4 to inhibit LTM is not dependent on deacetylase activity.

### HDAC4 co-localizes with MEF2 in Kenyon cells

**Figure 5:**
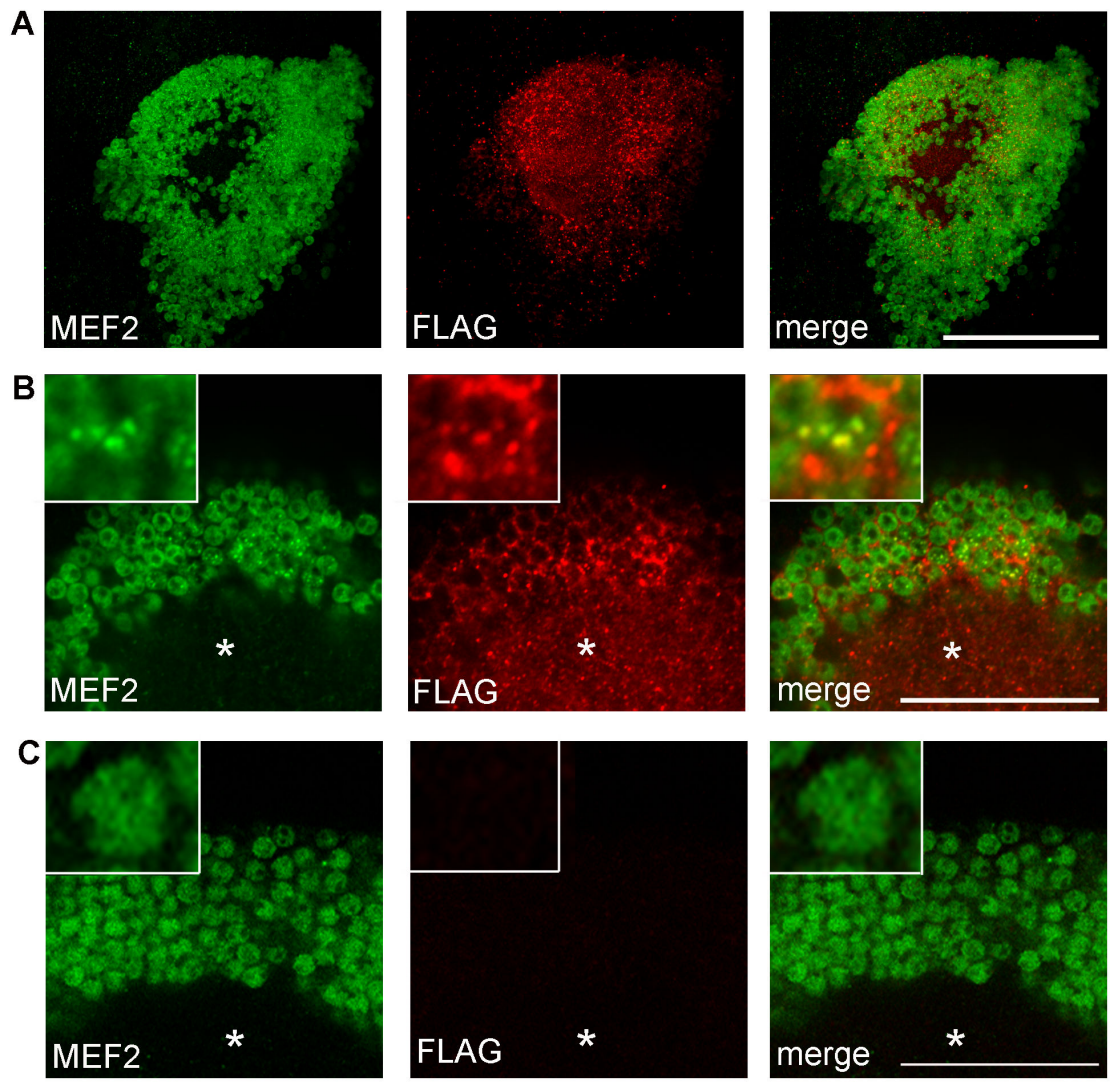
HDAC4 induces relocation of MEF2 in nuclei. A. Confocal projection through the posterior brain showing expression of MEF2 (green) and HDAC4 (red) in Kenyon cells. Scale bar = 50 μm. B. A single confocal plane (1 μm) through the Kenyon cells at the level of the calyx (asterisk) shows MEF2 is localized to the nuclei whereas HDAC4 is predominantly localized to the calyx and cytoplasm. In a subset of cells, nuclear HDAC4 expression is observed, which always co-localizes with MEF2. Inset shows a single cell body with punctate nuclear HDAC4 surrounded by a cytoplasmic halo of HDAC4. MEF2 has been recruited from a relatively even nuclear pattern to the punctate distribution. Scale bar = 25 μm. Inset is at 5.4x magnification. C. In control brains, MEF2 is distributed evenly across the nucleus and punctate localization is not observed, thus overexpression of HDAC4 has caused a redistribution of MEF2 from a relatively even nuclear pattern to a more punctate distribution. Scale bar = 25 μm. Inset is at 5.4x magnification.

The observation that HDAC4 does not require its enzymatic activity to suppress LTM led us to examine other mechanisms by which HDAC4 might regulate memory. It is well established that HDAC4 binds to and represses the transcriptional activity of MEF2, and this binding is independent of enzymatic activity [27,62]. MEF2 is expressed in Kenyon cells – indeed the enhancer element in the MB247 driver is derived from the MEF2 locus, and notably the MB expression pattern driven by MB247 is reminiscent of that of NP1617. We found that MEF2 and HDAC4 also co-localize in Kenyon cells (Figure 5A, B). In all nuclei in which punctate HDAC4 staining was present, the localization of MEF2 was redistributed from a relatively even distribution in the nucleus to co-localization with HDAC4 in punctate nuclear bodies (Figure 5B). In nuclei in which no HDAC4 was observed, MEF2 was localized in a more regular pattern without punctate staining. Similarly in control brains in which HDAC4 was not overexpressed, MEF2 was localized relatively evenly in nuclei and nuclear bodies were not seen (Figure 5C). 

### Knockdown of HDAC4 impairs LTM

**Figure 6:**
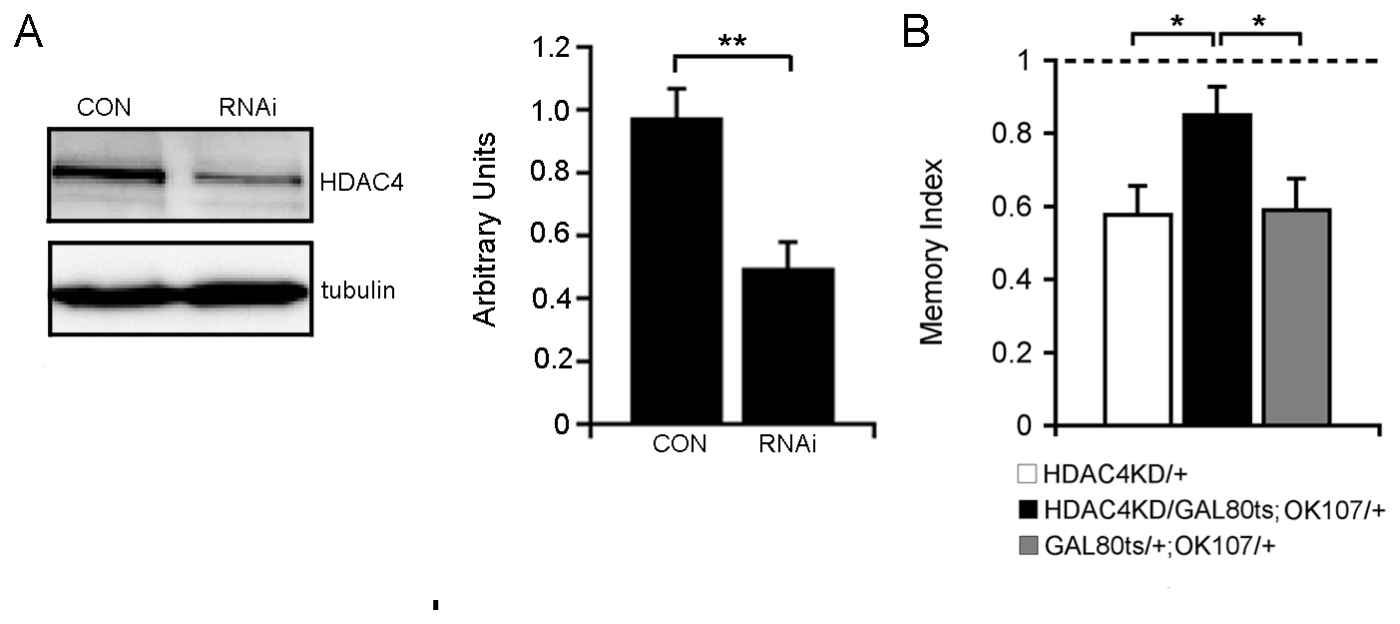
Knockdown of HDAC4 impairs LTM. HDAC4-RNAi expression was restricted to the adult brain with the TARGET system. A. Expression of HDAC4-RNAi in the fly brain repressed HDAC4 expression to ~50% of that of control brains. Western blot shows the expression of HDAC4::YFP in control (HDAC4::YFP/elav^C155^) and HDAC4 RNAi knockdown (HDAC4::YFP/elav^C155^; HDAC4RNAi/+) fly head lysates (labeled CON and RNAi, respectively). Blots were probed with anti-GFP to detect HDAC4::YFP and with anti-α-tubulin as a loading control. Image J was used to quantify band intensity (n=4 samples from 4 independent crosses, Student’s t-test, two-tailed, unpaired, **p<0.01). B. LTM was significantly impaired by OK107-GAL4 mediated knockdown of HDAC4 (HDAC4KD) (ANOVA, post-hoc Tukey’s HSD, *p<0.05).

Lastly, after establishing that HDAC4 is a negative regulator of memory, we postulated that decreasing the amount of HDAC4 would release the constraint on memory and result in improved LTM scores. However, on the contrary, RNAi-mediated knockdown of HDAC4 to ~50 % of wild-type (Figure 6A) resulted in impairment of LTM (Figure 6B), which suggests that wild-type levels HDAC4 are required for normal formation of LTM. 

## Discussion

Here, we provide evidence that HDAC4 plays an integral role in the regulation of LTM in Drosophila. We examined the impact of both increasing and decreasing brain-specific expression of HDAC4 on memory, in an identical genetic background. We have previously shown Rpd3 is required for LTM [14], and here we identify that a second HDAC also modulates LTM, as observed by an impairment in LTM following RNAi-mediated knockdown of HDAC4.

The MB, an important site for formation of both olfactory and courtship memory, was identified as a site of HDAC4 expression and analysis of the subcellular localization of FLAG-tagged HDAC4 in the MB revealed both cytoplasmic and nuclear localization. HDAC4 was detected in the MB lobes, which are the regions of synaptic output from the MB, but additionally receive input from extrinsic sources such as the dorsal paired medial neurons [63]. We also observed HDAC4 in the calyx, which constitutes the dendritic field of the Kenyon cells, and in the cytoplasm surrounding the Kenyon cell nuclei. Of the Kenyon cells that expressed HDAC4, only a subset displayed detectable nuclear localization of HDAC4. This varying localization is similar to that observed in the hippocampus of the mouse brain, in which nuclear localization of HDAC4 is observed in only a proportion of cells in the CA1, CA3 pyramidal regions and dentate gyrus, and is more commonly extranuclear [22]. HDAC4 nucleocytoplasmic shuttling is responsive to various physiological cues, including calcium influx [15], and the differences in subcellular localization may be reflective of variations in basal synaptic activity of the neurons.

Overexpression of HDAC4 resulted in a specific impairment in LTM, without altering one-hour memory. This is not attributable to a non-specific disruption of cellular function in Kenyon cells, as STM, which is dependent on an intact MB [41] was normal. By comparing memory phenotypes resulting from expression of HDAC4 with GAL4 drivers specific to MB subtypes, we determined that overexpression of HDAC4 in the γ lobe was likely responsible for the memory impairment. It has become increasingly evident that the three MB subtypes are not only structurally, but also functionally distinct and the specific roles they play in phases of memory continue to be refined [49,54,64,65]. Elucidation of the molecular processes that underlie long-term courtship memory is still in the early stages, however the γ lobe has been identified as a site at which modifications that underlie courtship memory occur. Deletion of the N-terminus of the cytoplasmic polyadenylation element–binding protein Orb2 results in a specific impairment in LTM formation, and restoration of Orb2 in the γ neurons during or immediately after a training session is sufficient to rescue this LTM deficit [49]. The Class I HDAC Rpd3 (HDAC1) is also required for long-term suppression of courtship behavior. Knockdown of Rpd3 in the MB impairs courtship LTM, but not STM [14]. No phenotype is observed with the α/β neuron-specific driver c739, suggesting that Rpd3 is required in the α'/β' and/or γ neurons. Whether Rpd3 and HDAC4 interact during formation of courtship LTM is not yet known, however overexpression of HDAC4 did not result in obvious colocalization of Rpd3 and HDAC4 in punctate nuclear bodies, suggesting that HDAC4 is not recruiting Rpd3 into a transcriptional repressor complex.

The transcriptional activator CREB plays an essential role in formation of protein-synthesis-dependent memory across the animal kingdom [66] and in Drosophila, CREB has been shown to be required for formation of long-term olfactory and courtship memory [64,67,68]. Expression in γ neurons of CREB2-b, which encodes a repressor isoform of CREB, impairs spaced long-term olfactory conditioning [64]. A Drosophila CREB2 activator also enhances long-term courtship memory [68], but it has yet to be determined if this effect is mediated through specific MB subtypes. CREB immunoprecipitates with HDAC4 in mouse cerebellar extracts, and this interaction is enhanced in mice homozygous null for ataxia telangiectasia mutated (*Atm* ^*-/-*^), resulting in a neurodegenerative phenotype in which HDAC4 is predominantly nuclear [69]. It will be of interest to investigate whether one mechanism by which HDAC4 regulates LTM is repression of CREB, through either transcriptional repression or direct binding. Expression of a CaMKII-targeted hairpin RNA in γ neurons also specifically impairs spaced long-term olfactory conditioning [64]. CaMKII is well established to play several important roles in memory formation, which are mediated through its kinase activity as well as by binding to various proteins at the post-synaptic density (for review see 70). As CaMKII has been demonstrated to mediate nuclear export of HDAC4 via phosphorylation [15,23], we hypothesize that regulation of HDAC4 subcellular localization may be an additional function of CaMKII in LTM. 

We found that a catalytically impaired mutant of HDAC4 impairs LTM, which is consistent with a mouse study in which brain-specific expression of a nuclear-restricted mutant of HDAC4 (lacking a catalytic domain) also prevented spatial memory [33]. How does overexpression of HDAC4 impair memory? In cultured cortical neurons, nuclear-restricted HDAC4 represses a set of genes that is enriched for many with known synaptic functions [33]. Conversely, MEF2 induces expression of genes involved in synaptic development and function in both primary hippocampal cultures, and in mouse brains following exploration of a novel environment [71]. HDAC4 associates with MEF2 on chromatin and comparison of genome-wide mRNA profiling studies revealed that a subset of the genes that are activated by MEF2 are also repressed by HDAC4 [33]. These data suggest that HDAC4 may prevent memory formation, at least in part, by suppression of MEF2 target gene expression and it may thus be hypothesized that during memory formation, activity-induced exit of HDAC4 from the nucleus relieves the repression of MEF2, allowing expression of plasticity genes. In support of this hypothesis, we found that overexpression of HDAC4 in the fly brain impaired memory, and this was associated with colocalization of HDAC4 and MEF2 in nuclei. 

Lending further support to this theory, a brain specific knockout of MEF2 in the mouse hippocampus impairs contextual fear conditioning, with an associated reduction in excitatory synapses [31], suggesting that MEF2 is plays an essential role in memory. However, it has also been reported that MEF2 activity is down-regulated during memory formation and expression of a constitutively active form of MEF2 in the mouse hippocampus and amygdala blocks both spatial and fear memory, respectively [32], indicating that MEF has the capacity to negatively regulate memory. Clearly the role of MEF2 in memory formation is complex and context dependent. The relationship between HDAC4 and MEF2 is also intricate; HDAC4 binds to MEF2, preventing it from activating transcription. However for HDAC4 to enter the nucleus, it requires binding to MEF2, thus, in effect, MEF2 escorts its own repressor [47]. This opens up the possibility that the impairment of memory by overexpression of MEF2 [32] may be in part due to increased HDAC4 in the nucleus. How the interplay between these proteins is coordinated to regulate memory formation is intriguing and the focus of future research.

While overexpression of HDAC4 impaired LTM, we also found that, in the same genetic background, knockdown of HDAC4 also resulted in a deficit in LTM. In mouse, a brain-specific knockout of HDAC4 causes impairment of both LTP and hippocampal-dependent memory [34]. This is unlikely the result of a developmental deficit as HDAC4 was not knocked out until postnatal day ten, and furthermore, conditional knockout of HDAC4 in cortical and hippocampal neurons, or in neural progenitors has no obvious effect on neuronal structure or viability in the mouse [72]. We found that HDAC4 was predominantly extra-nuclear, localizing to the cytoplasm, axons, and dendritic field, and in mouse, scanning electron microscopy has revealed a concentration of HDAC4 at the postsynaptic density [22]. Whether HDAC4 is required in nucleus, extra-nuclear cellular compartments and/or synapse for normal LTM formation is yet to be determined – in fact to date there is little evidence of a non-nuclear role for HDAC4 in any brain function. Interestingly, data from a recent study is suggestive of a neuroprotective function for cytoplasmic HDAC4; in *Atm* ^*-/-*^ mice, which display neuronal degeneration and behavioural impairments, HDAC4 accumulates in neuronal nuclei and represses MEF2 and CREB target genes. Viral vector–mediated expression of a cytoplasmic restricted form of HDAC4 is sufficient to reverse the neurodegeneration and resulted in significant improvement in motor behaviour [69]. These data suggest that prevention of HDAC4 nuclear entry may be a plausible therapeutic strategy, and identification of cytoplasmic binding partners and/or potential non-histone deacetylase targets will be critical in order to shed light on the non-nuclear role of HDAC4.

## Materials and Methods

### Fly Strains

All flies were cultured on standard medium on a 12 hour light/dark cycle and maintained at a temperature of 25°C unless otherwise indicated. Canton S flies were used as wild-type controls. *w* ^*^ *; P*{*w+mW.hs=GawB*}*OK107* (OK107-GAL4), *y1w67c23; P*{*w+mW.hs=GawB*}*c*739 (c739-GAL4), *w* ^1118^;*P*{*w+mW.hs=GawB*}*c305a* (c305a-GAL4), *w* ^1118^;*P*{*w+mW.hs=GawB*}*1471* (1471-GAL4), *y* ^*1*^ *w* ^*^ *; P*{*UAS-mCD8::GFP.L*}*LL5* (UAS-CD8::GFP), and *w* ^1118^ *; P*{*w[+mC*]*=UAS-RedStinger*}*6* (UAS-nls.dsRED) were obtained from the Bloomington Drosophila Stock Center. *y* ^*^ *w* ^*^ *P*{*w[+mW.hs*]*=GawB*}*HDAC4^NP1617^/FM7c* (NP1617-GAL4) and *y* ^*^ *w* ^*^ *P*{*w[+mW.hs*]*=GawB*}*HDAC4^NP3558^/ FM7c* (NP3558-GAL4) were obtained from the Kyoto Stock Center. *w* ^*^ *; P*{*w+mC=tubP-GAL80ts*}*10* (tubP-GAL80^ts^), p{MEF2-GAL4.247}(MB247-GAL4) and *w*(*CS10*) strains were kindly provided by R. Davis (The Scripps Research Institute, Jupiter, FL). The *w* ^1118^ *; P*{*GD9446*}*v20522* strain (HDAC4RNAi) was obtained from the Vienna Drosophila RNAi Center.

To generate the UAS-HDAC4OE strain, a full length HDAC4 cDNA (clone GH08881, transcript variant D, inserted into pOT2) was obtained from the BDGP Gold Collection, Drosophila Genome Research Center. Sequencing of the cDNA revealed a 1 nucleotide deletion at position 427, which resulted in a frame shift and premature truncation of the protein. To replace the missing nucleotide, site-directed mutagenesis was performed with the Quikchange II Site Directed Mutagenesis Kit (Agilent Technologies, Santa Clara, CA, USA) using the following primers: Forward 5' Cgcaattcctggaaagcgccatgtgaattcaccgc 3' and Reverse 5' GCGGTGAATTCACATGGCGCTTTCCAGGAATTGCG 3'. The GH08881 clone was fully sequenced to confirm that a cytosine residue had been inserted and that no new mutations had been introduced. To fuse an N-terminal FLAG epitope tag to HDAC4, the N-terminal fragment of the corrected GH08881 clone was PCR amplified with a forward primer containing the FLAG sequence. The primer sequences were as follows: FLAGfor 5' ATTAGATATCCAACATGGACTACAAGGACGACGACGATGACAAGTCTAGTCCCGACGATAGA 3' and FLAGrev 5' TCAGAGGCGATATGGATCCGATCTGCTGATCGATATCCAC 3'. A 188 bp product containing the FLAG-tag and an *Eco*RV linker was amplified with Expand Polymerase (Roche), digested with *Eco*RV and inserted into *Eco*RV digested pOT2/HDAC4, resulting in the addition of an in frame N-terminal FLAG-tag. pOT2/FLAG-HDAC4 was digested with *Bgl*II and *Xho*I to release FLAG-HDAC4. This was cloned into *Bgl*II and *Xho*I of pUASTattB. The resulting plasmid was microinjected into embryos of the *y w, P*{*hs-flp*}*; P*{*3xP3-RFP=attP-86F*}*; P*{*3xP3-RFP=phic-31{3xP3-GFP=vas-phic31*}}*102F* strain containing an attP landing site on the third chromosome. The attP strain and pUASTattB plasmid were obtained from Konrad Basler, University of Zurich, [73]. 

The HDAC4 H968A mutant was generated by site directed mutagenesis of pOT2/FLAG-HDAC4 with the following primers: H986Afor 5' CGGCCGCCGGGCCATGCCGCGGAGGAGGCCAA 3' and H968Arev 5' TTGGCCTCCGCGGCATGGCCCGGCGGCCG 3'. Following mutagenesis, FLAG-HDAC4-H986A was fully sequenced to confirm the presence of the mutation and absence of any new mutations. pOT2/FLAG-HDAC4 H968A was digested with *Bgl*II and *Xho*I and cloned into *Bgl*II and *Xho*I of pUASTattB. Transgenic flies were generated by GenetiVision (Houston, Texas, USA), using the P2 injection strain (attP insertion site 3L68A4).

All strains were outcrossed for a minimum for five generations to *w*(*CS10*) flies. A homozygous line harbouring *w*(*CS10*)*; P*{*w+mC=tubP-GAL80ts*}*10* and *P*{*w+mW.hs=GawB*}*OK107* (tubP-GAL80_ts_;OK107-GAL4) was generated by standard genetic crosses, as was (tubP-GAL80_ts_,c739-GAL4); (tubP-GAL80_ts_,1471-GAL4); (tubP-GAL80_ts_; MB247-GAL4) and (tubP-GAL80_ts_, c305a-GAL4).

### Behavioral Analyses

The repeat training courtship assay [41,49,74] was used to assess memory. The premise of this assay is that male flies learn that they have been previously rejected by a female. Once a female fly has mated, she will rebuff the advances of male flies for an extended period of time. A virgin male fly will court vigorously when presented with a female, however his behavior can be modified by her continued rejection, such that when presented with a second mated female or an immobilized virgin, his effort at courtship are reduced. This phenomenon is termed courtship suppression and can be used to reliably assess both short- and long-term associative memory [41,49–52]. 

A one-hour training session has been shown to produce a strong short-term memory that decays after two to three hours when tested with an anaesthetized virgin [74] and by eight hours when tested with a mobile mated female [49]. Memory that lasts longer than 30 minutes after a one hour training session is dependent on an intact mushroom body [41], however an earlier phase of courtship memory (0 - 30 mins) is thought to be independent of the mushroom body, as mushroom body-ablated flies display normal immediate recall after a one hour training session [41]. During a training session of five to eight hours duration, a male will engage in multiple bouts of courtship with a mated female with breaks in between. These repeated attempts at mating are thought to represent the repetition that is required for consolidation of some types of LTM [75,76] and result in formation of a robust LTM [41,49,77–79] that has been shown to persist at least five to seven days after training [41,78,79]. The methodology has been previously described in detail [14].

Male flies to be tested were collected and housed in single vials for 4 - 6 days. For each experiment, control genotypes were tested at the same time as those expressing the knockdown or overexpression construct. In all experiments, the scorer was blind to the genotype of the flies. All naïve and trained groups contained (n=15 to 25) males. All experiments were performed under ambient light. For experiments using the TARGET system [80], the temperature was modulated by placing flies at the permissive temperature of 19°C (GAL80ts active) or the restrictive temperature of 30°C (GAL80ts inactive), as appropriate. For induction of transgene expression, flies were transferred to 30°C three days prior to training to allow maximum GAL4-mediated expression of the UAS construct. Flies were trained at 30°C in an incubator under white light and remained at 30°C until 30 minutes before testing, at which time they were transferred to 25°C for equilibration to the testing conditions. 

A courtship index (CI) is calculated as the percentage of the ten-minute period spent in courtship behavior. In order to compare memory across genotypes, a memory index (MI) was calculated by dividing the courtship index (CI) of each test fly by the mean CI of the sham flies of that genotype (CI_test_/mCI_sham_) [50–52]. A score of 0 indicates the highest memory performance possible, and a score ≥1.0 indicates no memory. For statistical analyses, data was arcsine transformed in order to approximate a normal distribution and significance was assessed by one-way ANOVA with post-hoc Tukey’s HSD test. When comparing only two genotypes, the student’s t-test (two-tailed, unpaired) was used. The significance level was set at P<0.05.

### Immunohistochemistry

Whole flies were fixed in PFAT/DMSO (4% paraformaldehyde in 1X PBS +0.1% Triton X-100+5% DMSO) for one hour then washed in 1xPBS. Brains were microdissected in 1xPBS then post fixed in PFAT/DMSO for 20 mins and stored in MeOH at -20°C. Following rehydration in PBT (1xPBS+0.5% triton X-100) brains were blocked in immunobuffer (5% normal goat serum in PBT) for two hours at room temperature. They were then incubated overnight at room temperature with primary antibody (mouse anti-FLAG, 1:5000 (Sigma), rabbit anti-FLAG 1:1000 (Sigma), mouse anti-Trio 1:200 (DSHB), rabbit anti-Drosophila Rpd3 (Abcam ab1767, 1:1,000), rabbit anti-GFP (Abcam ab290, 1:20,000), rabbit anti-MEF2 (H.T. Nguyen, 1:1,500) then incubated overnight at 4°C with secondary antibody (goat anti-mouse Alexa555, or goat–anti-rabbit Alexa488, Molecular Probes, 1:200) and mounted with Antifade. The monoclonal antibody anti-Trio (94A clone) was obtained from the Developmental Studies Hybridoma Bank developed under the auspices of the NICHD and maintained by The University of Iowa, Department of Biology, Iowa City, IA 52242. For confocal microscopy, optical sections were taken with a Leica TCS SP5 DM6000B Confocal Microscope. Image stacks were taken at intervals of 1 μm (whole brain) or 0.5 μm (mushroom body) and processed with Leica Application Suite Advanced Fluorescence (LAS AF) software. 

### Western Blotting

50 - 100 flies were collected in 15 ml tubes and frozen in a dry ice/ethanol bath. The tubes were vortexed to snap the heads from the bodies, and then the heads were separated and collected on a piece of acetate over dry ice. Cytoplasmic extracts were prepared by homogenizing heads in 50 μl of Buffer I (1 mM DTT, 0.1 mM EDTA, 15 mM HEPES pH 7.6, 10 mM KCl, 5 mM MgCl_2_, 0.5 mM EGTA, 0.35 M sucrose) with a disposable mortar and pestle, then centrifuging at 7,700 g for 15 minutes at 4°C. The supernatant was retained as the cytoplasmic fraction. Following protein quantification with a BCA kit, 20 μg of each sample was loaded onto a 10% SDS-PAGE gel and resolved at 200V. Protein was transferred onto nitrocellulose and blocked for >1 hour in 5% skim milk powder in TBST (50 mM Tris, 150 mM NaCl, 0.05% Tween-20, pH7.6). The membrane was incubated overnight at 4°C in primary antibody and one hour in secondary antibody. Antibodies used were anti-GFP (ab290, Abcam, 1:4000) and anti-α-tubulin (12G10 clone, Developmental Studies Hybridoma Bank, 1:500), detection was performed with ECL Select (GE). For quantification of HDAC4 knockdown, four samples were analyzed (from four separate fly crosses over two separate blots). Band intensities were quantified using Image J and normalized to tubulin. Statistical significance was assessed by one-way ANOVA with post-hoc Tukey’s HSD test. 

## Supporting Information

Figure S1 **YFP-tagged HDAC4 is weakly expressed in the MB.** A. Western blot showing detection of HDAC4::YFP. Cytoplasmic brain lysates were prepared of all samples. Left panel: Anti-GFP detects cytoplasmic GFP when expressed by elav-GAL4 in neuronal nuclei. Right panel: A product migrating at ~160-170 kDa is detected in HDAC4::YFP brain lysate but not controls (left and middle lane). HDAC4::YFP is predicted to be 153-163 kDa (www.flybase.org). B. Brains from HDAC4::YFP (HDAC4::YFP/*w*[CS10]) and control (w[CS10]) flies were processed for immunohistochemistry with anti-GFP. A single confocal plane (1 μm) is shown through each image. Confocal settings were identical between control and HDAC4::YFP brains. B,C. MB-specific expression was not observed in any control brains. The staining around the outside of the brain is non-specific and reflects the high gain setting required to detect the weak HDAC4::YFP signal. D,E. HDAC4::YFP expression is weakly detected in the mushroom body (white arrows). Scale bar = 100 μm. F, H. Magnification of the MB in control brains in B and C. Scale bar = 50 μm. G, I. Magnification of the MB in HDAC4::YFP brains in D and E. Scale bar = 25 μm. CON: *w*(CS10); HDAC4: HDAC4::YFP/*w*(CS10).
(TIF)

Figure S2 **Expression of FLAG-tagged HDAC4 in the MB lobes.** OK107-GAL4-mediated expression of HDAC4 was induced in adult brains with the TARGET system at 30°C for three days. Whole-mount brains were processed for immunohistochemistry with anti-FLAG (red) and anti-FasII (green, expressed in α/β and γ lobes) or anti-Trio (green, expressed in α'/β' and γ lobes). A. HDAC4 colocalizes with FasII in the α lobe. B. Minimal to no expression in the α' lobe was usually observed as shown by minimal colocalization of HDAC4 and Trio. Scale bar = 25 μm (A and B). C. HDAC4 also colocalizes with FasII in the β lobe. D. No colocalization of HDAC4 with Trio was seen in the β' lobe. E. HDAC4 co-localizes with FasII in the γ lobe. Scale bar = 50 μm (C-E).
(TIF)

Figure S3 **FLAG-tagged HDAC4 is not expressed at 19°C.** Flies harbouring OK107-GAL4, tub-GAL80ts and UAS-FLAG-HDAC4 were raised and maintained at 19°C as a control to examine the efficacy of the TARGET system at inhibiting transgene expression at 19°C. Whole-mount brains were processed for immunohistochemistry with anti-FLAG (red) and anti-Rpd3 (green). Rpd3 is expressed in the majority of neuronal nuclei and assists in visualization of general anatomy of the brain. A. Frontal confocal projection through one hemisphere of the brain. Scale bar = 100 μm. B. Posterior confocal projection through one hemisphere of the brain. Scale bar = 100 μm.
(TIF)
