# Supplementary figures and images for: The Histone Deacetylase HDAC4 Regulates Long-Term Memory in Drosophila
Source: PLoS One. 2013 Dec 9;8(12):e83903. doi: 10.1371/journal.pone.0083903 (PMC3857321; doi:10.1371/journal.pone.0083903)

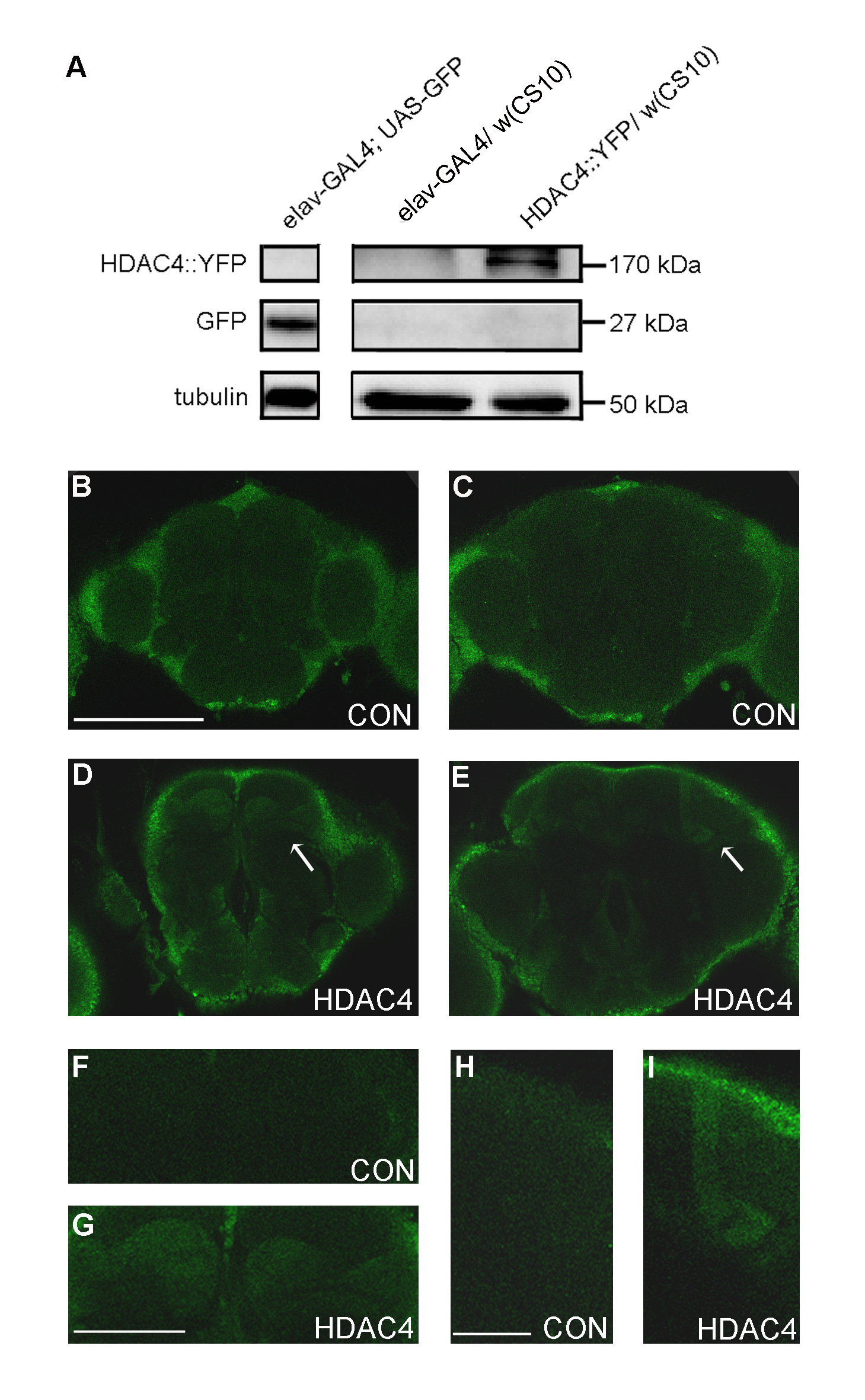

Supplement: Figure S1 — YFP-tagged HDAC4 is weakly expressed in the MB. A. Western blot showing detection of HDAC4::YFP. Cytoplasmic brain lysates were prepared of all samples. Left panel: Anti-GFP detects cytoplasmic GFP when expressed by elav-GAL4 in neuronal nuclei. Right panel: A product migrating at ~160-170 kDa is detected in HDAC4::YFP brain lysate but not controls (left and middle lane). HDAC4::YFP is predicted to be 153-163 kDa (www.flybase.org). B. Brains from HDAC4::YFP (HDAC4::YFP/w[CS10]) and control (w[CS10]) flies were processed for immunohistochemistry with anti-GFP. A single confocal plane (1 μm) is shown through each image. Confocal settings were identical between control and HDAC4::YFP brains. B,C. MB-specific expression was not observed in any control brains. The staining around the outside of the brain is non-specific and reflects the high gain setting required to detect the weak HDAC4::YFP signal. D,E. HDAC4::YFP expression is weakly detected in the mushroom body (white arrows). Scale bar = 100 μm. F, H. Magnification of the MB in control brains in B and C. Scale bar = 50 μm. G, I. Magnification of the MB in HDAC4::YFP brains in D and E. Scale bar = 25 μm. CON: w(CS10); HDAC4: HDAC4::YFP/w(CS10). (TIF) [file pone.0083903.s001.tif]

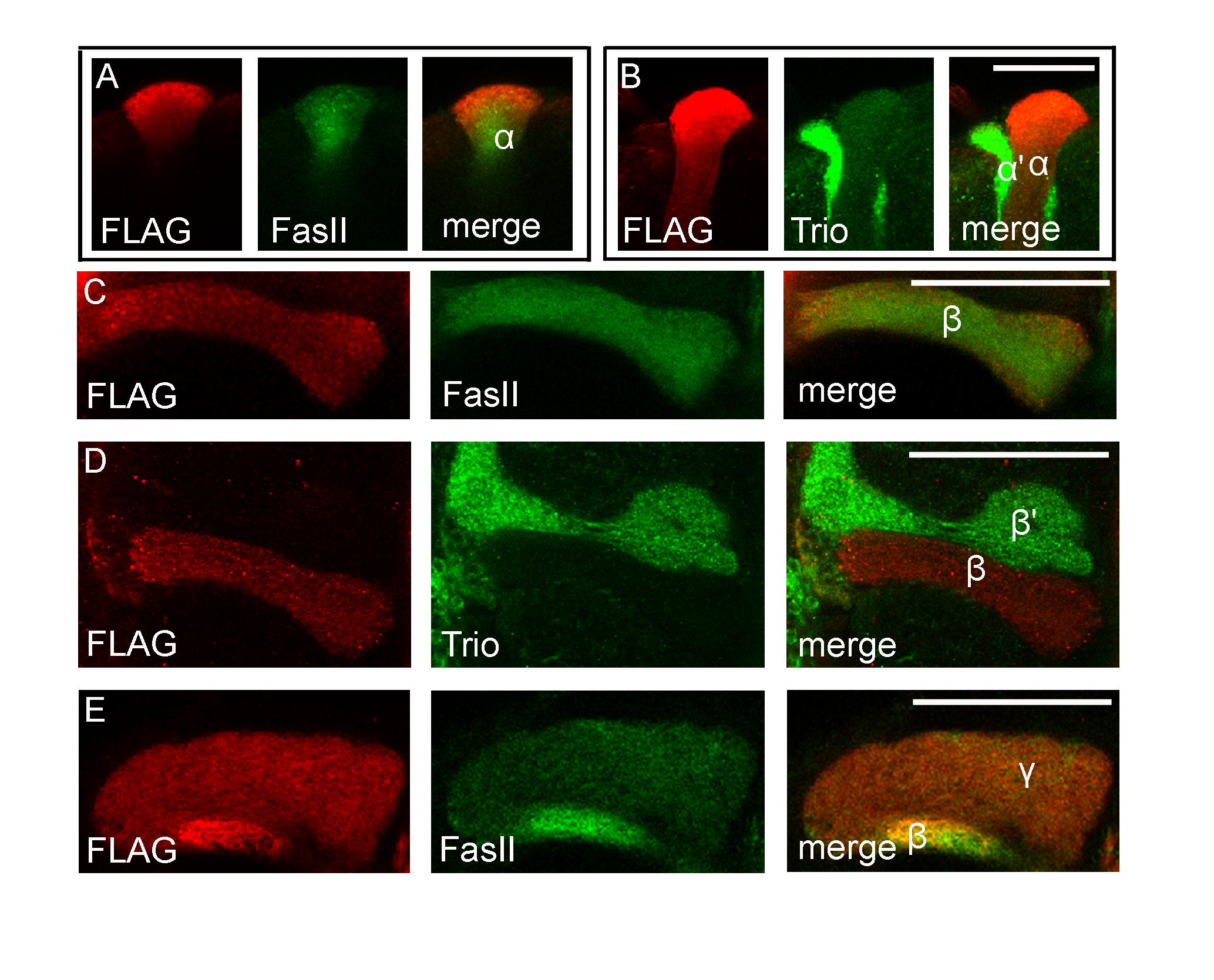

Supplement: Figure S2 — Expression of FLAG-tagged HDAC4 in the MB lobes. OK107-GAL4-mediated expression of HDAC4 was induced in adult brains with the TARGET system at 30°C for three days. Whole-mount brains were processed for immunohistochemistry with anti-FLAG (red) and anti-FasII (green, expressed in α/β and γ lobes) or anti-Trio (green, expressed in α'/β' and γ lobes). A. HDAC4 colocalizes with FasII in the α lobe. B. Minimal to no expression in the α' lobe was usually observed as shown by minimal colocalization of HDAC4 and Trio. Scale bar = 25 μm (A and B). C. HDAC4 also colocalizes with FasII in the β lobe. D. No colocalization of HDAC4 with Trio was seen in the β' lobe. E. HDAC4 co-localizes with FasII in the γ lobe. Scale bar = 50 μm (C-E). (TIF) [file pone.0083903.s002.tif]

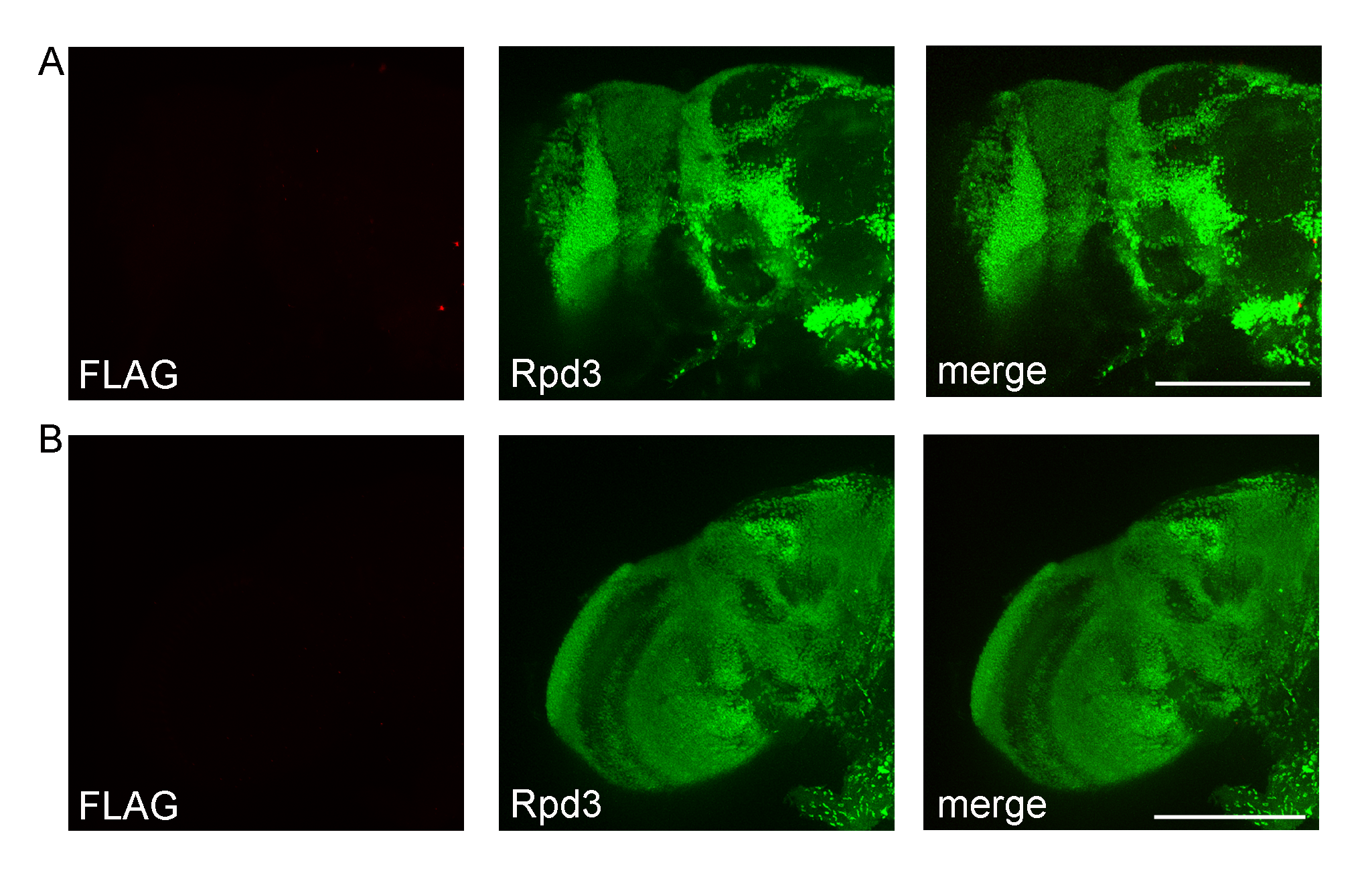

Supplement: Figure S3 — FLAG-tagged HDAC4 is not expressed at 19°C. Flies harbouring OK107-GAL4, tub-GAL80ts and UAS-FLAG-HDAC4 were raised and maintained at 19°C as a control to examine the efficacy of the TARGET system at inhibiting transgene expression at 19°C. Whole-mount brains were processed for immunohistochemistry with anti-FLAG (red) and anti-Rpd3 (green). Rpd3 is expressed in the majority of neuronal nuclei and assists in visualization of general anatomy of the brain. A. Frontal confocal projection through one hemisphere of the brain. Scale bar = 100 μm. B. Posterior confocal projection through one hemisphere of the brain. Scale bar = 100 μm. (TIF) [file pone.0083903.s003.tif]
